# Noninvasive Intracranial Compliance Monitoring Following Hand‐Applied Photobiomodulation and Ultrasound in Fibromyalgia: A Pilot Study

**DOI:** 10.1002/jbio.70390

**Published:** 2026-11-10

**Authors:** Matheus Henrique Camargo Antonio, Tiago Zuccolotto Rodrigues, Ana Carolina Negraes Canelada, Fernanda Mansano Carbinatto, Vanessa Garcia, Vanderlei Salvador Bagnato, Antonio Eduardo de Aquino Junior

**Affiliations:** ^1^ Instituto de Ensino e Pesquisa (IEP), Irmandade da Santa Casa de Misericórdia de São Carlos São Carlos São Paulo Brazil; ^2^ Physics Institute of São Carlos, University of São Paulo São Carlos São Paulo Brazil; ^3^ Pos‐Graduation Program of Biotechnology Federal University of São Carlos São Carlos São Paulo Brazil; ^4^ Department of Biomedical Engineering Texas A&M University College Station Texas USA

**Keywords:** brain monitoring, central sensitization, chronic pain, fibromyalgia, intracranial compliance, neurophysiology, photobiomodulation, therapeutic ultrasound

## Abstract

Fibromyalgia (FM) is associated with chronic pain and altered neurophysiological regulation. This exploratory pilot study examined intracranial pulse waveform parameters and clinical outcomes following combined photobiomodulation therapy (PBMT) and therapeutic ultrasound (TUS) applied bilaterally to the hands. Twenty women participated (FM, *n* = 10; healthy comparators, *n* = 10); both groups received 10 sessions over 5 weeks. Noninvasive measurements included waveform morphology, P2/P1 ratio, and time to peak. Pain intensity, Fibromyalgia Impact Questionnaire, and Pittsburgh Sleep Quality Index scores were also assessed. At baseline, participants with FM showed a higher proportion of P2 > P1 waveforms and longer time to peak than healthy participants. Following treatment, the FM group showed fewer P2 > P1 waveforms, shorter time to peak, and lower pain, functional impact, and sleep disturbance scores. The P2/P1 ratio did not change significantly. Without a sham‐treated group, these findings indicate preliminary associations rather than treatment efficacy or causality.

## Graphical abstract

In women with fibromyalgia, combined photobiomodulation and therapeutic ultrasound applied to the hands was associated with improvements in pain, sleep quality, and functional impact, alongside changes in intracranial pulse waveform parameters. These preliminary findings support further investigation of peripheral biophysical stimulation and its potential relationship with intracranial physiology.

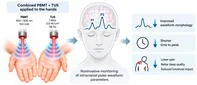

## 1 Introduction

Fibromyalgia (FM) is a complex chronic pain syndrome characterized by widespread musculoskeletal pain, persistent fatigue, sleep disturbances, and significant functional impairment [1, 2]. Central sensitization is currently considered one of the main pathophysiological mechanisms underlying the syndrome, involving abnormal amplification of nociceptive processing at both spinal and supraspinal levels [3, 4]. In addition to chronic pain, patients with FM frequently present neuroautonomic alterations, cognitive dysfunction, and neuropsychiatric manifestations, suggesting a condition of systemic neurophysiological dysregulation. Epidemiologically, FM affects approximately 2% to 4% of the global population, with a predominance in females [1, 2]. The interaction between chronic pain, anxiety, and depression contributes to a positive neuroendocrine and autonomic feedback cycle that is often difficult to manage clinically [5].

Recent advances in functional and molecular neuroimaging [3, 4] have reinforced central sensitization as an important pathophysiological mechanism underlying FM. This state of hyperexcitability involves dysfunction of descending inhibitory and facilitatory pain pathways, as well as dysregulation of neurotransmitters such as serotonin, norepinephrine, and substance P. Emerging evidence also suggests the involvement of neuroimmunological mechanisms and the gut–brain axis in the modulation of pain and neuroinflammation associated with FM [6, 7].

Among the cardinal symptoms of FM, sleep disturbances are highly prevalent and act as important perpetuating factors of the syndrome. Polysomnographic studies have demonstrated relevant alterations in sleep architecture, including reduced slow‐wave sleep (N3) and alpha‐wave intrusion during deep sleep [8, 9]. These alterations impair mechanisms related to descending pain modulation and neuroendocrine balance, contributing to amplification of pain perception and functional deterioration. In parallel, functional neuroimaging studies [9, 10, 11] have demonstrated hyperactivation of brain regions involved in affective‐emotional processing and pain modulation, including the amygdala, anterior insula, and anterior cingulate cortex.

Depressive and anxiety symptoms frequently coexist in FM and are associated with greater pain intensity, poorer quality of life, and increased functional impairment [4, 12]. Evidence suggests that these manifestations share pathophysiological mechanisms related to neuroendocrine dysregulation, neuroinflammation, and alterations in central neural circuits involved in pain and emotional processing [13, 14, 15].

Recently, intracranial pressure regulation and intracranial compliance (ICC) have received attention in the investigation of chronic pain and related conditions [16, 17]. ICC describes the capacity of the intracranial compartment to accommodate changes in volume with relatively small changes in pressure and is an important component of intracranial physiology [18]. Although central sensitization is well established in fibromyalgia, a direct relationship between ICC alterations and its clinical manifestations has not been established. Preliminary investigations have also explored combined biophysical interventions in conditions characterized by pain and functional symptoms, including growing pains [19] and post‐COVID‐19 sequelae [20]. These observations provide an exploratory rationale for assessing noninvasive intracranial pulse waveform parameters in fibromyalgia, without assuming that they represent a diagnostic marker of the syndrome.

Photobiomodulation therapy (PBMT) uses red and near‐infrared light to influence biological responses associated with cellular metabolism and signaling [21, 22]. Therapeutic ultrasound (TUS) produces acoustic mechanical effects and, depending on the exposure conditions, thermal effects that may influence local tissue responses [23, 24, 25]. Because these modalities act through different physical mechanisms, their combined application warrants investigation; however, additive or synergistic effects cannot be assumed without evaluating the modalities separately [21, 24].

The palmar region was selected on the basis of previous clinical reports describing the application of combined PBMT and TUS to the hands in fibromyalgia [20, 22]. The hands provide an accessible and reproducible peripheral treatment site with extensive sensory innervation. Peripheral stimulation could plausibly be associated with central responses through somatosensory and autonomic pathways or circulating signaling mechanisms, but these pathways were not directly investigated in the present study. Thus, palmar application represents a previously explored intervention approach rather than a demonstrated superior site for influencing intracranial physiology.

Previous reports using the photosonic Recupero system (MMOptics, Brazil) have described clinical observations following combined PBMT and TUS in individuals with fibromyalgia [26, 27]. More broadly, studies of nonpharmacological interventions for chronic pain and the neurobiology of fibromyalgia highlight the complexity of peripheral and central contributions to symptom modulation [28, 29]. Nevertheless, whether palmar PBMT and TUS are associated with changes in intracranial pulse waveform parameters remains uncertain. Therefore, this exploratory pilot study aimed to characterize noninvasive intracranial pulse waveform parameters in women with fibromyalgia compared with healthy participants and to examine changes in these parameters, pain intensity, sleep quality, and functional impact following combined PBMT and TUS applied to the hands.

## 2 Materials and Methods

### 2.1 Ethical Aspects and Study Setting

The present study was conducted at the Photodynamic Therapy Clinic Unit of Santa Casa Hospital of São Carlos, under the coordination of the Physics Institute of São Carlos (IFSC), University of São Paulo (USP). The research protocol was approved by the Brazilian National Research Ethics Committee (CONEP) under CAAE number 13789319.5.0000.8148, in accordance with Resolution 466/2012. All participants were enrolled after signing an informed consent form, and all procedures were conducted in accordance with the principles of the Declaration of Helsinki.

### 2.2 Equipment and Combined Intervention

Therapeutic interventions were performed using the RECUPERO system (MMOptics, São Carlos, Brazil; ANVISA registration no. 80051420029), a device developed by the Technical Support Laboratory of IFSC‐USP (Patent no. BR102014007397‐3 A2). This device (Figure 1) enables simultaneous delivery of photobiomodulation therapy (PBMT) and therapeutic ultrasound (TUS) to the same treatment region. Although both modalities were applied together, the present study did not assess their separate contributions or test for additive or synergistic interactions.

**FIGURE 1:**
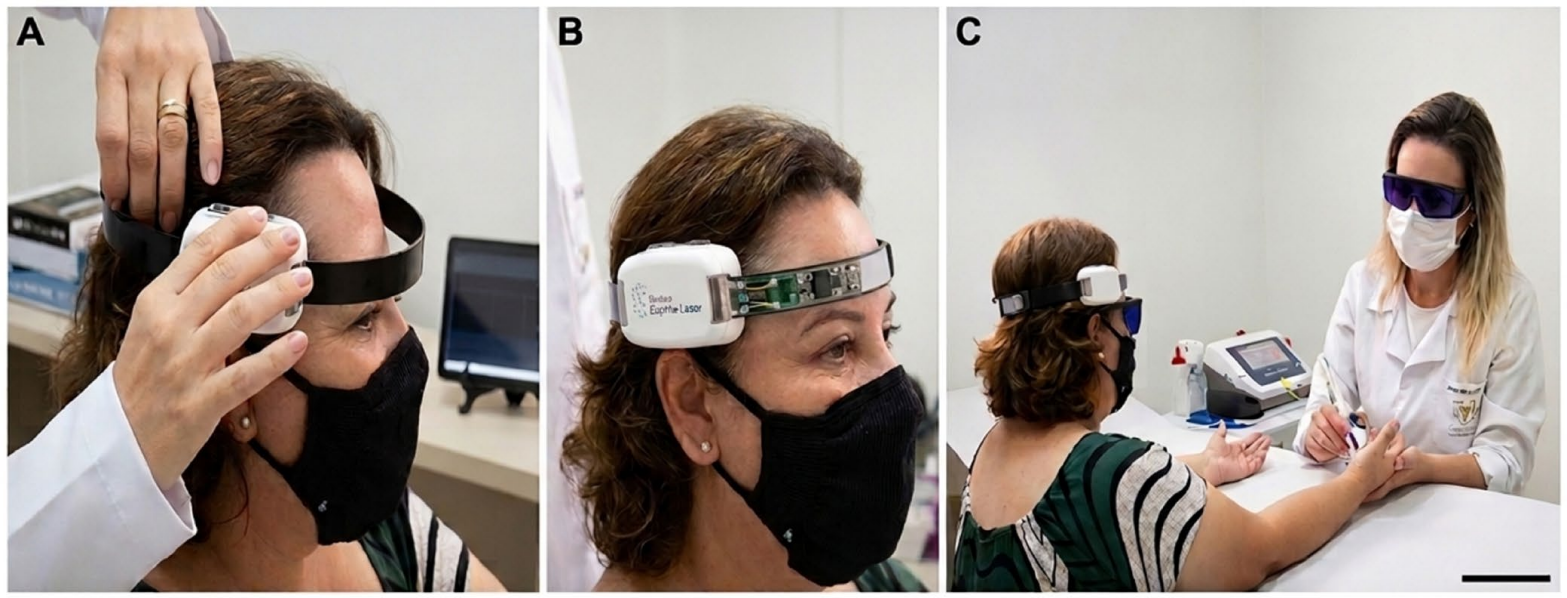
Experimental protocol and noninvasive intracranial pulse waveform monitoring during combined photobiomodulation therapy and therapeutic ultrasound. (A–B) Placement of the Braincare sensor over the temporoparietal region. (C) Bilateral palmar application of PBMT and TUS using the RECUPERO system. Participants completed 10 sessions, twice weekly over five consecutive weeks. Waveform measurements were acquired during baseline, intervention, and recovery.

### 2.3 Patient Selection and Experimental Protocol

The sample consisted of 20 female participants aged between 20 and 80 years, divided into two groups: the Fibromyalgia Group (*n* = 10) and the Healthy Control Group (*n* = 10). This study was designed as a pilot, exploratory, and proof‐of‐concept investigation, without formal sample size calculation, aiming to identify preliminary physiological and clinical signals associated with the intervention and to estimate parameters for future controlled studies. The healthy control group was included to establish a physiological reference for intracranial compliance and to enable comparison with the patterns observed in patients with fibromyalgia. Therefore, this group was not designed as a therapeutic placebo control, but rather as a physiological comparator within an exploratory pilot design. The study was not structured as a randomized controlled clinical trial, but as an exploratory investigation focused on physiological characterization and response to intervention. The diagnosis of FM was previously established through specialized medical evaluation, based on compatible clinical history and recognized diagnostic criteria for the syndrome, including widespread pain, chronic fatigue, reduced muscle strength, sleep disturbances, headache, gastrointestinal disorders, and neuropsychiatric symptoms. Individuals with a history of neoplasia, pregnancy, open wounds, local infections, hematomas, cardiac pacemakers, or metallic prostheses at the application site were excluded from the study. Continuation of pharmacological treatment previously prescribed by the participants' attending physicians was allowed for both groups.

### 2.4 Dosimetry and Therapeutic Procedure

The intervention was applied bilaterally to the palmar regions over 10 sessions, twice weekly for five consecutive weeks. This schedule was informed by a previous frequency‐comparison investigation by our group in fibromyalgia, which evaluated 2, 3, 5, and 10 sessions per week while maintaining a total of 10 sessions. Twice‐weekly treatment showed favorable clinical responses and was selected as a feasible previously investigated schedule, without assuming that it was optimal. Both groups received the same combined PBMT and TUS protocol.

1. Photobiomodulation therapy (PBMT) was delivered at 660 nm (red) and 808 nm (near‐infrared), with a nominal output power of 100 mW per emitter.
2. Therapeutic ultrasound (TUS) was delivered in pulsed mode at 1 MHz, with a reported intensity of 0.8 W/cm^2^ and a pulse repetition frequency of 48 Hz.

PBMT and TUS were delivered simultaneously for 5 min per application point. The monitoring protocol allocated 10 min to the intervention period. Additional device‐specific parameters required for complete dose reporting, including laser beam area and ultrasound duty cycle and effective radiating area, should be verified against the equipment records before inclusion; they cannot be established from the original manuscript alone.

### 2.5 Clinical Assessments and Data Collection Instruments

Participants were evaluated at baseline and following completion of the 10‐session protocol. Pain intensity was also recorded immediately before and after each session. Pain was assessed using a 10‐cm Visual Analogue Scale (VAS), ranging from 0 (no pain) to 10 (worst imaginable pain). Fibromyalgia impact was evaluated using the Fibromyalgia Impact Questionnaire (FIQ), which assesses functional and symptom‐related burden over the preceding 7 days; total scores range from 0 to 100, with higher scores indicating greater impact. Sleep quality was assessed using the Portuguese‐language Pittsburgh Sleep Quality Index (PSQI), which covers seven sleep‐related components over the preceding month; global scores range from 0 to 21, with higher scores indicating poorer sleep quality. The Portuguese‐language questionnaires used for data collection are provided as Files S1 (FIQ) and S2 (PSQI), subject to permission for reproduction.

### 2.6 Intracranial Compliance (ICC) Monitoring

Intracranial pulse waveform morphology was monitored noninvasively using the BcSs‐PICNI‐2000 system (Braincare Health Technology, São Carlos, Brazil; ANVISA registration no. 81157910002). The system detects micrometric cranial bone deformation associated with intracranial pressure pulsations using high‐sensitivity strain gauges. Waveform‐derived measures were used as exploratory indicators of intracranial dynamics; this method does not directly measure absolute intracranial pressure or establish a diagnosis of intracranial hypertension.

### 2.7 Positioning and Data Acquisition

The sensor was positioned over the frontotemporal‐parietal region (Figure 1), approximately 5 to 6 cm above the external auditory meatus, at the temporoparietal transition area. The device was secured using an adjustable cranial band, applying minimal mechanical pressure to ensure adequate sensor coupling to the scalp without interfering with cranial expansibility. Signals were digitized, filtered, and processed using the Braincare software platform (updated version), allowing automated analysis of ICP waveform morphology.

### 2.8 Waveform Analysis and Monitoring Protocol

Intracranial pulse waveform analysis included the percussion peak (P1), associated primarily with arterial pulsation, and the tidal peak (P2), whose relative prominence may reflect aspects of intracranial pressure–volume dynamics. The P2/P1 ratio, the proportions of P2 ≥ P1, P2 ≈ P1, and P2 ≤ P1 waveform patterns, and time to peak were analyzed as exploratory physiological measures. These waveform features were not interpreted as standalone diagnostic measures of intracranial compliance impairment. Participants were monitored in a seated position over a 20‐min acquisition period: baseline (5 min), simultaneous PBMT and TUS intervention (10 min), and recovery (5 min).

### 2.9 Statistical Analysis

Data were expressed as mean ± standard deviation (SD). Data normality was assessed using the Kolmogorov–Smirnov test. Paired Student's *t*‐test was used for intragroup comparisons between baseline and postintervention time points. Unpaired Student's *t*‐test was applied for intergroup comparisons between healthy individuals and patients with fibromyalgia. Due to the pilot nature of the study and the reduced sample size, statistical findings were interpreted in an exploratory manner. In addition to *p*‐values, percentage differences and observed physiological trends were also considered in the interpretation of the findings. Statistical analyses were performed using Statistica 10.0 software (StatSoft Inc., Tulsa, Oklahoma, USA), adopting a significance level of *p* < 0.05.

## 3 Results

Figure 2 presents the comparison of intracranial compliance before and after intervention. At the preintervention time point, healthy individuals showed predominance of the P2 ≈ P1 pattern (53.4%), indicative of normal physiological condition, followed by P2 > P1 (37.9%) and P2 < P1 (8.4%). In contrast, patients with fibromyalgia exhibited predominance of the P2 > P1 pattern (70.9%), suggesting altered intracranial compliance, followed by P2 ≈ P1 (24.2%) and P2 < P1 (4.9%). Comparison between groups revealed a statistically significant difference for the P2 > P1 pattern (A vs. G, *p* < 0.05).

**FIGURE 2:**
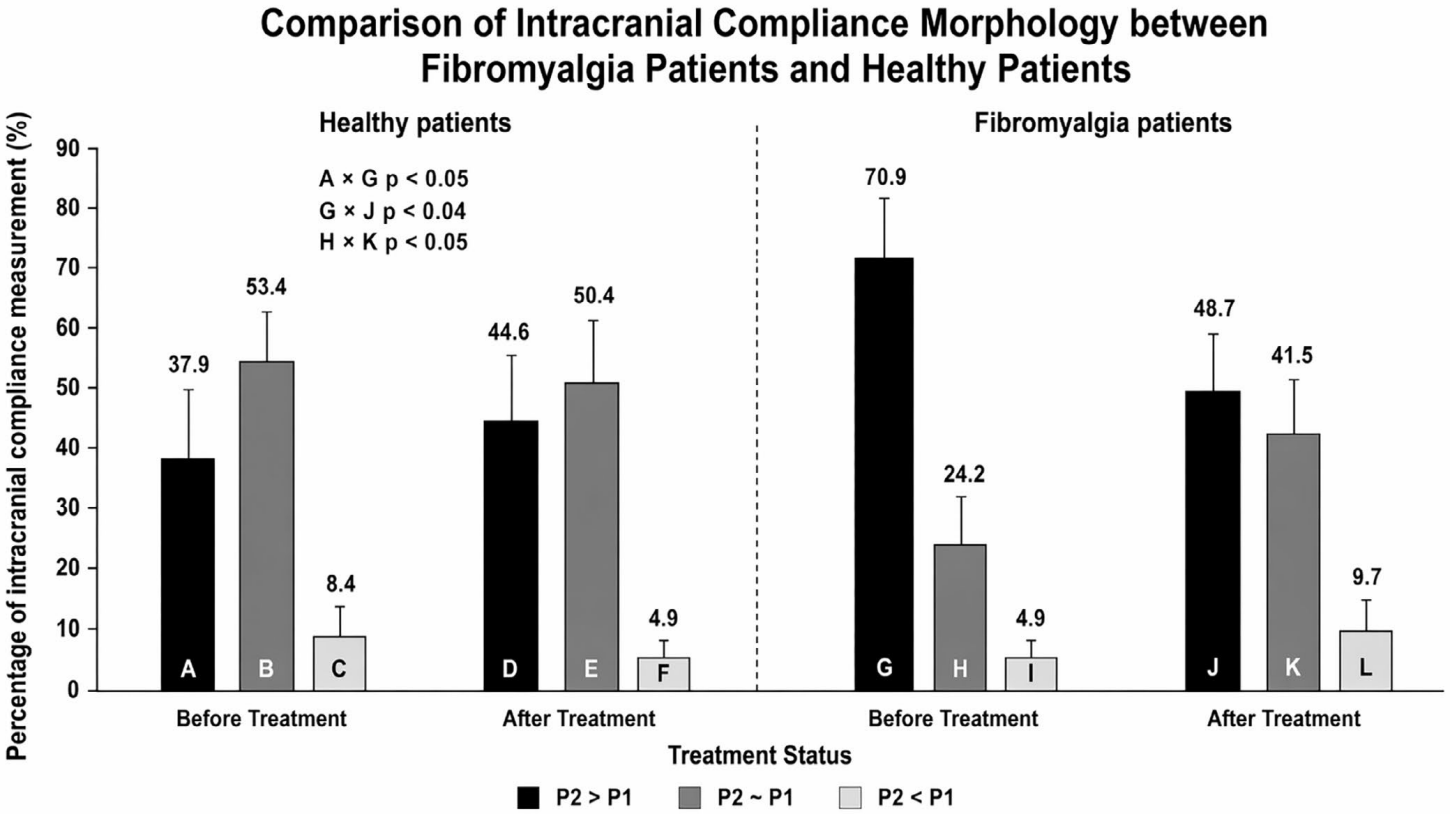
Comparison of intracranial compliance waveform morphology between healthy individuals and patients with fibromyalgia before and after synergistic PBMT + TUS intervention. Intracranial compliance waveform distribution was categorized as P2 > P1, P2 ≈ P1, and P2 < P1 patterns. Healthy individuals exhibited predominance of the physiological P2 ≈ P1 pattern at baseline, whereas patients with fibromyalgia showed higher prevalence of the altered P2 > P1 morphology, suggestive of reduced intracranial compliance. Following intervention, the fibromyalgia group demonstrated a significant reduction in the occurrence of the P2 > P1 pattern and an increase in the P2 ≈ P1 pattern. Statistical significance: A versus G, *p* < 0.05; G versus J, *p* < 0.04; H versus K, *p* < 0.05.

After intervention using the systemic method based on the synergistic application of laser and ultrasound over the palms of the hands, no statistically significant alterations were observed in the control group (healthy individuals). In contrast, the fibromyalgia group demonstrated a significant reduction in the occurrence of the P2 > P1 pattern (G vs. J, *p* < 0.04), decreasing from 70.9% to 48.7%. In parallel, a significant increase in the P2 ≈ P1 pattern was observed (H vs. K, *p* < 0.05), rising from 24.2% to 41.5%, accompanied by a relative reduction in the predominance of the P2 > P1 pattern and approximation to the profile observed in the healthy group.

Figure 3 presents the comparison of the P2/P1 ratio between healthy individuals and patients with fibromyalgia before and after synergistic laser and ultrasound intervention. At baseline, a statistically significant difference was observed between groups (A vs. C, *p* < 0.02), with higher mean values in the fibromyalgia group (1.35) compared with the healthy group (1.14), suggesting altered intracranial compliance dynamics.

**FIGURE 3:**
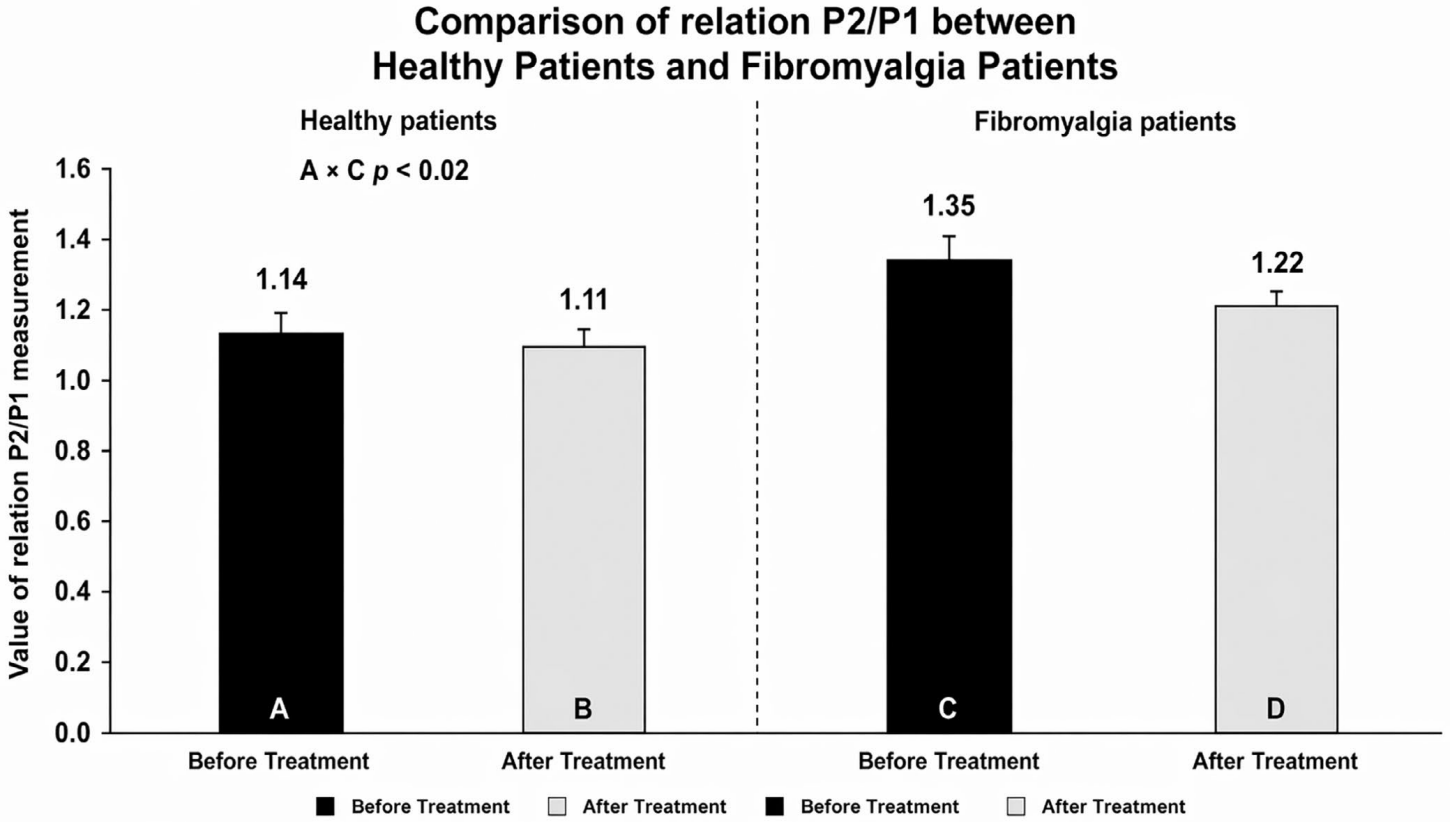
Comparison of the P2/P1 ratio between healthy individuals and fibromyalgia patients before and after synergistic PBM + UST intervention. Baseline analysis demonstrated significantly higher P2/P1 values in fibromyalgia patients compared with healthy controls, indicating altered intracranial compliance dynamics. Although no statistically significant differences were observed after intervention, a reduction trend in the P2/P1 ratio was identified in the fibromyalgia group. The P2/P1 parameter was interpreted as an exploratory complementary marker, while waveform morphology and *time to peak* showed statistically significant alterations in the comparisons between groups and time points. Statistical significance: A versus C, *p* < 0.02.

After intervention, no statistically significant differences were identified between pre‐ and posttreatment time points in either group. However, a percentage reduction in the P2/P1 ratio of 2.63% was observed in the healthy group and 9.63% in the fibromyalgia group, indicating a tendency toward improvement despite the absence of statistical significance. Therefore, the isolated P2/P1 ratio was considered an exploratory complementary parameter, whereas P2 > P1 and P2 ≈ P1 waveform patterns, as well as the *time to peak* parameter, demonstrated statistically significant alterations associated with group comparisons and evaluated time points.

Figure 4 presents the comparison of the *time to peak* parameter between healthy individuals and patients with fibromyalgia before and after intervention. In the healthy group, no statistically significant differences were observed between the evaluated time points (0.21 vs. 0.20). In contrast, the fibromyalgia group demonstrated a significant reduction after intervention (C vs. D, *p* < 0.05), with values decreasing from 0.27 at the preintervention time point to 0.22 after intervention.

**FIGURE 4:**
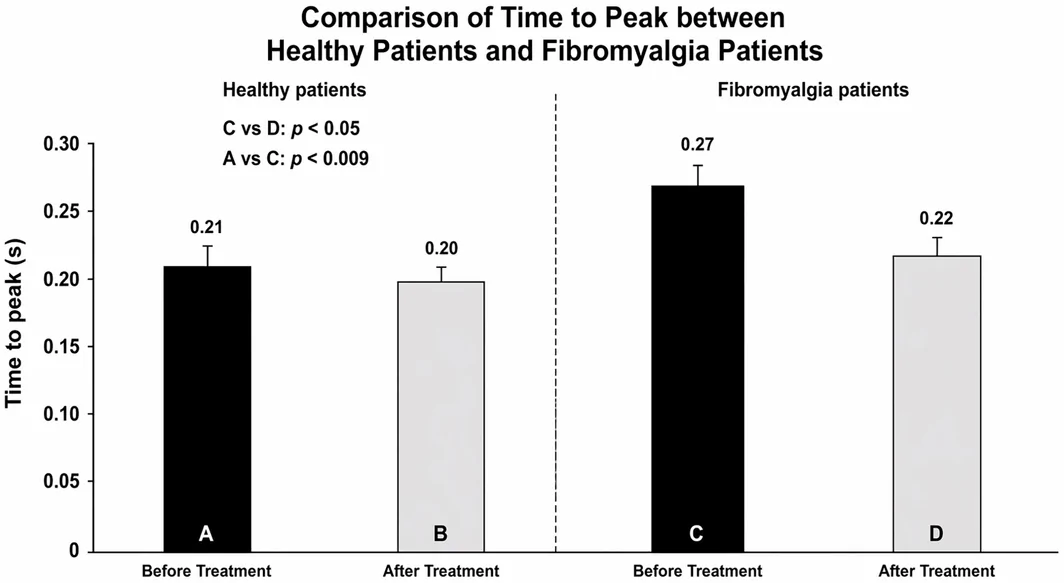
Comparison of the *time to peak* parameter between healthy individuals and fibromyalgia patients before and after synergistic PBM + UST intervention. No significant differences were observed in healthy individuals between pre‐ and postintervention periods. In contrast, fibromyalgia patients exhibited a significant reduction in *time to peak* values following intervention, suggesting modulation of intracranial compliance dynamics. Baseline values were significantly higher in fibromyalgia patients than in healthy controls, indicating altered physiological behavior associated with the syndrome. Following intervention, values approached those observed in healthy participants. Statistical significance: C versus D, *p* < 0.05; A versus C, *p* < 0.009.

Additionally, comparison between groups at baseline (A vs. C) revealed a statistically significant difference (*p* < 0.009), with higher values observed in the fibromyalgia group, indicating an initial alteration in this parameter. Following intervention, values in the fibromyalgia group approached those observed in the healthy group.

Figure 5 presents the comparison of Fibromyalgia Impact Questionnaire (FIQ) scores between healthy individuals and patients with fibromyalgia before and after intervention. In the healthy group, a statistically significant reduction in scores was observed after intervention, decreasing from 35 to 19 points (*p* < 0.03). Similarly, the fibromyalgia group also demonstrated a significant reduction, with scores decreasing from 64 to 38 points (*p* < 0.001).

**FIGURE 5:**
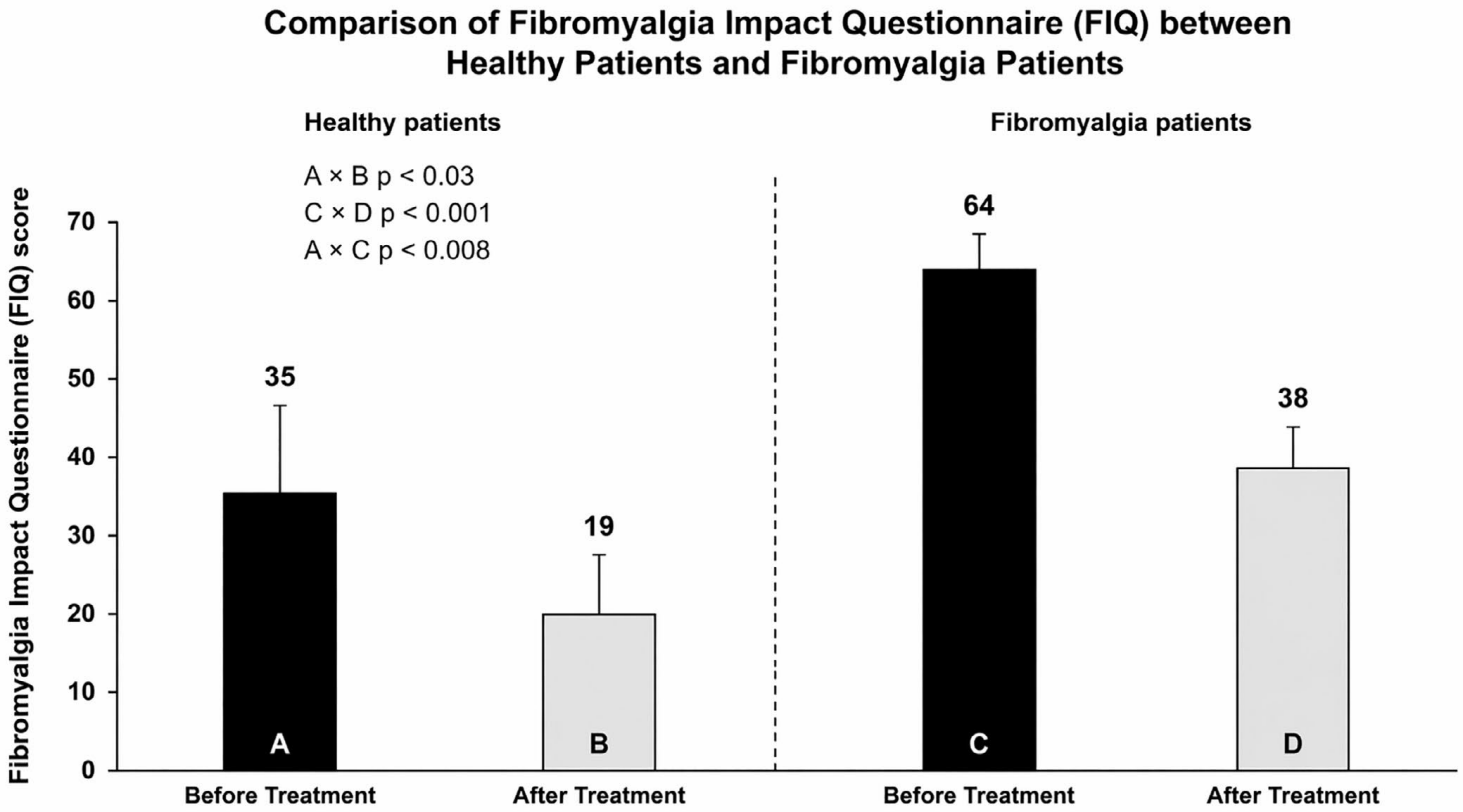
Effects of synergistic PBM + UST intervention on Fibromyalgia Impact Questionnaire (FIQ) scores in healthy individuals and fibromyalgia patients. Both groups demonstrated significant reductions in FIQ scores following intervention, with a more pronounced improvement observed in fibromyalgia patients. Postintervention values in the fibromyalgia group approached baseline values observed in healthy individuals, with marked reduction in FIQ scores after intervention. Statistical significance: Healthy group, *p* < 0.03; fibromyalgia group, *p* < 0.001.

Additionally, postintervention values in the fibromyalgia group (38 points) approached baseline values observed in the healthy group (35 points), accompanied by a marked reduction in functional impairment scores after intervention.

Figure 6 presents the comparison between healthy individuals and patients with fibromyalgia regarding the Visual Analogue Scale (VAS) before and after intervention. In the healthy group, a statistically significant reduction in pain scores was observed, decreasing from 2.3 to 0.8 (A vs. B, *p* < 0.04). Similarly, the fibromyalgia group demonstrated a significant reduction, with scores decreasing from 7.3 to 2.5 (C vs. D, *p* < 0.0001).

**FIGURE 6:**
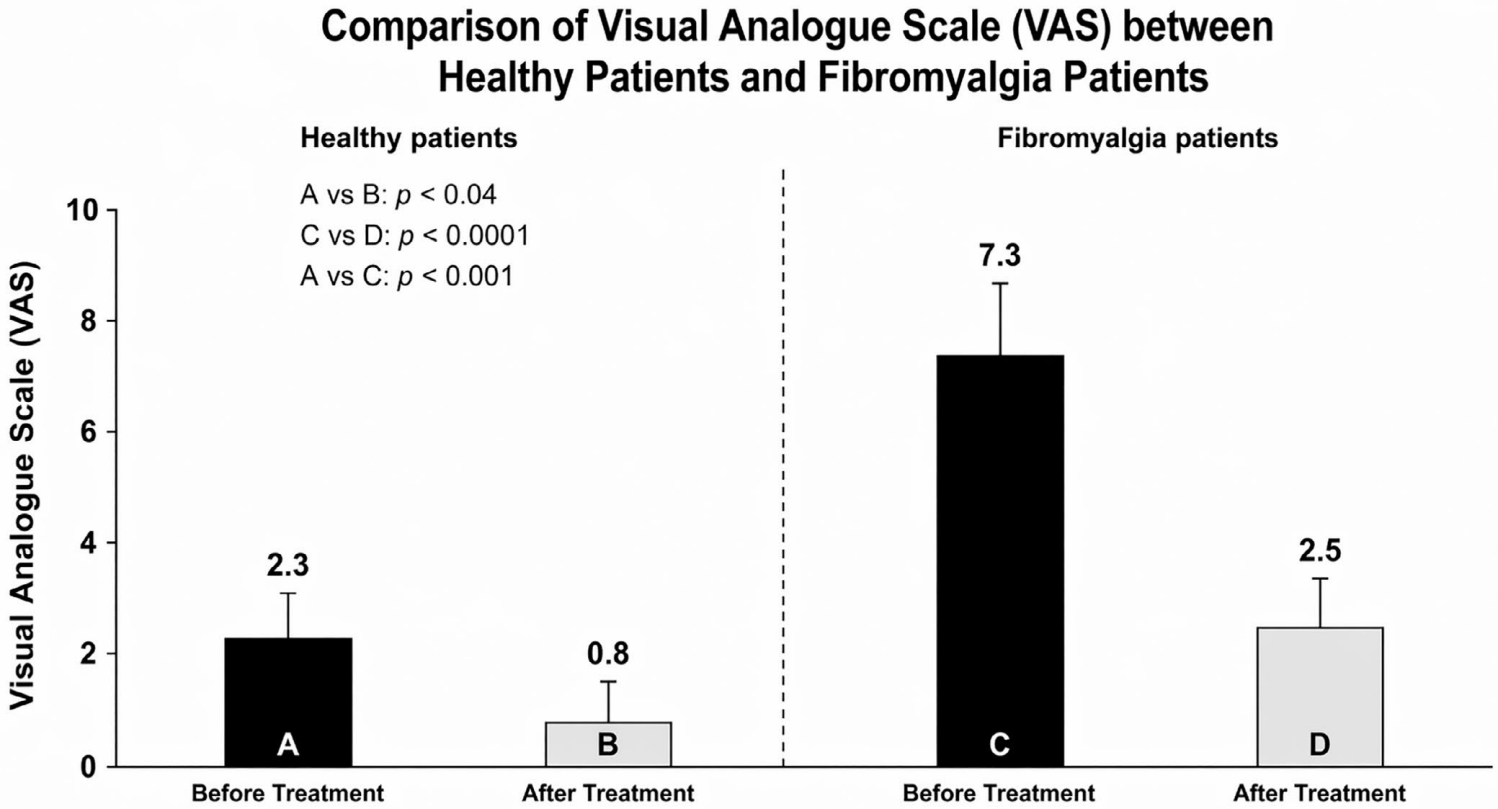
Effects of synergistic PBM + UST intervention on pain intensity measured by the Visual Analogue Scale (VAS). Fibromyalgia patients exhibited significantly higher baseline pain scores compared with healthy controls. Following intervention, both groups demonstrated significant reductions in pain intensity, with marked improvement observed in the fibromyalgia group. Postintervention pain levels in fibromyalgia patients approached baseline levels observed in healthy participants, with significant reduction in VAS scores after intervention. Statistical significance: A versus B, *p* < 0.04; C versus D, *p* < 0.0001; A versus C, *p* < 0.001.

Comparison between groups at baseline (A vs. C) revealed a statistically significant difference (*p* < 0.001), with higher pain levels observed in the fibromyalgia group. Additionally, postintervention values in the fibromyalgia group (2.5) approached baseline values observed in the healthy group (2.3), accompanying the significant reduction in pain scores after intervention.

Figure 7 presents the comparison of sleep quality, assessed using the Pittsburgh Sleep Quality Index (PSQI), between healthy individuals and patients with fibromyalgia before and after intervention. In the healthy group, a statistically significant reduction in PSQI scores was observed, decreasing from 7.8 to 3.5 (A vs. B, *p* < 0.005). Similarly, the fibromyalgia group also demonstrated a significant reduction, with scores decreasing from 10.8 to 6.7 (C vs. D, *p* < 0.004).

**FIGURE 7:**
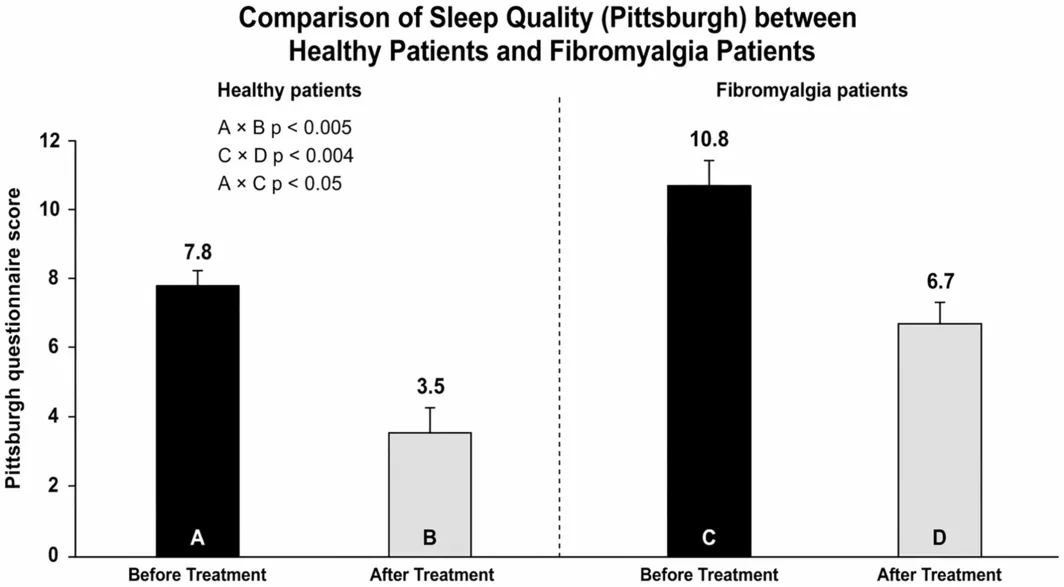
Effects of synergistic PBM + UST intervention on sleep quality assessed by the Pittsburgh Sleep Quality Index (PSQI). Fibromyalgia patients presented significantly poorer sleep quality at baseline compared with healthy individuals. After intervention, significant reductions in PSQI scores were observed in both groups, indicating improved sleep quality. Postintervention values in fibromyalgia patients were lower than baseline values observed in healthy controls, with significant reduction in PSQI scores after intervention. Statistical significance: A versus B, *p* < 0.005; C versus D, *p* < 0.004; A versus C, *p* < 0.05.

Comparison between groups at baseline (A vs. C) revealed a statistically significant difference (*p* < 0.05), with higher scores observed in the fibromyalgia group, indicating poorer sleep quality compared with healthy individuals. Following intervention, the value observed in the fibromyalgia group (6.7) was lower than the baseline value observed in the healthy group (7.8), demonstrating a reduction in scores associated with poorer sleep quality.

## 4 Discussion

Fibromyalgia (FM) remains one of the most challenging conditions in current clinical practice, not only because of its high prevalence, but also due to the complexity of its pathophysiology, which is centered on mechanisms of central sensitization and systemic neurobiological dysregulation. Evidence indicates that nociceptive amplification, associated with autonomic and neuroendocrine dysfunctions, sustains a persistent state of chronic pain and functional impairment, directly affecting patients' quality of life [1, 2].

In this context, the present study explored whether noninvasive intracranial pulse waveform parameters could provide complementary physiological information in women with FM. At baseline, the FM group exhibited a higher proportion of P2 ≥ P1 waveforms than healthy participants. These findings suggest differences in waveform morphology between groups but do not establish a diagnostic role for intracranial compliance monitoring or demonstrate that intracranial pressure was elevated [16, 17].

Although the P2/P1 ratio decreased numerically in the FM group following the intervention, the change was not statistically significant. In contrast, time to peak decreased significantly, and the distribution of waveform morphologies shifted toward a lower proportion of P2 ≥ P1 patterns. These exploratory findings indicate changes in the measured waveform parameters over time but should not be interpreted as definitive evidence of normalization of intracranial physiology [18].

These findings are directly aligned with the central sensitization model, in which peripheral and central alterations coexist and mutually reinforce one another [16, 24]. Fibromyalgia may be understood as a condition of systemic neurophysiological dysregulation, in which sensory, autonomic, and neurovascular alterations coexist.

Clinically, this dysregulation is expressed through symptoms widely described in the literature, including widespread pain, persistent fatigue, sleep disturbances, and cognitive dysfunction, frequently associated with anxiety and depression [3, 4]. These symptoms do not occur in isolation, but rather coexist in an integrated manner, sustained by alterations in neural networks involved in pain modulation and emotional processing [9, 10, 11].

The use of validated instruments, including the Fibromyalgia Impact Questionnaire (FIQ), Visual Analogue Scale (VAS), and Pittsburgh Sleep Quality Index (PSQI), enabled a comprehensive evaluation of the clinical impact of the intervention. These instruments are widely recognized for their sensitivity in detecting changes in functionality, pain intensity, and sleep quality, which represent central dimensions of FM. In the present study, these measures demonstrated pain reduction and functional improvement, reinforcing the clinical relevance of the physiological findings.

Previous reports have described clinical outcomes following photobiomodulation and combined therapeutic approaches in individuals with FM [26, 27, 30, 31]. The present study extends these preliminary observations by examining noninvasive intracranial pulse waveform parameters alongside patient‐reported outcomes. However, the absence of a sham‐treated group prevents attribution of the observed changes specifically to the intervention.

Although treatment was applied to the palmar regions, a relationship between peripheral stimulation and central physiological responses remains biologically plausible but unconfirmed. The hands are richly innervated, and somatosensory input may influence central pain processing and autonomic regulation. Local photobiological and acoustic responses might also be associated with circulating signaling mediators. Neither neural transmission nor humoral mechanisms were measured in this study; consequently, these pathways remain hypotheses rather than established explanations for the observed findings.

Following the intervention, improvements were observed in clinical questionnaire scores and selected intracranial waveform parameters. The parallel direction of these changes is of exploratory interest, but the study did not establish correlations between individual clinical and physiological responses or determine whether they shared a common mechanism.

The impact of FM extends beyond pain and involves significant limitations in functionality, sleep quality, and mental health. The persistence of these symptoms is associated with a negative feedback cycle in which pain, fatigue, and sleep disturbances reinforce one another, thereby impairing therapeutic response [12, 15]. In this context, interventions capable of acting at multiple levels become particularly relevant.

The combined application of photobiomodulation and therapeutic ultrasound was based on the distinct biological effects previously described for each modality. Photobiomodulation has been associated with cellular metabolic regulation and inflammatory signaling, whereas therapeutic ultrasound produces mechanical effects that may influence local tissue responses [19, 20, 21, 22, 23]. However, the present study did not include photobiomodulation‐only or ultrasound‐only groups and therefore cannot determine whether the modalities produced additive or synergistic effects. In particular, enhanced light penetration or energy bioavailability due to ultrasound was not measured and should not be inferred. The findings should be interpreted as observations following a combined intervention rather than evidence of a specific interaction between modalities.

The primary physiological objective was to examine noninvasive intracranial pulse waveform parameters before and after combined palmar photobiomodulation and therapeutic ultrasound. Although the observed waveform changes may motivate further investigation of intracranial dynamics in FM, they do not demonstrate a direct treatment effect on intracranial pressure or establish intracranial compliance as a validated therapeutic biomarker [17, 18].

At baseline, women with FM exhibited a higher proportion of P2 ≥ P1 waveforms and longer time to peak than healthy participants. After the intervention, the FM group showed changes in these measures, including a reduction in time to peak. These findings warrant further study but cannot establish whether the changes reflect treatment‐specific modulation, natural variability, or other factors.

An additional consideration is the proposed relationship between fibromyalgia and idiopathic intracranial hypertension (IIH). Prior investigations by Hulens and colleagues and Ketenci and colleagues have explored possible overlaps involving intracranial pressure regulation and symptoms observed in FM [32, 33]. These reports provide a hypothesis that may warrant investigation when interpreting baseline differences in intracranial waveform morphology. However, intracranial compliance and intracranial pressure are distinct physiological parameters, and the waveform measurements used here cannot diagnose IIH. Cerebrospinal fluid opening pressure, optic nerve sheath diameter, and other diagnostic indicators of intracranial hypertension were not assessed. Therefore, this possible relationship cannot explain the observed changes with certainty and should be tested in future studies using appropriate neurological assessments.

From a physiological perspective, the relationship between peripheral palmar stimulation and intracranial physiological responses may involve both neural and circulating signaling pathways. Peripheral sensory stimulation could influence central somatosensory processing, pain‐modulatory circuits, and autonomic regulation, while local photobiological and mechanical responses might contribute to systemic signaling through circulating mediators and vascular regulatory mechanisms. These pathways could, in principle, be associated with cerebral perfusion, vascular regulation, or cerebrospinal fluid dynamics [18]. However, neural activity, autonomic function, circulating mediators, cerebral perfusion, and cerebrospinal fluid dynamics were not directly assessed. Their contributions therefore remain unknown, and a causal relationship between palmar stimulation and changes in intracranial waveform parameters cannot be established.

Taken together, the findings show concurrent changes in patient‐reported pain, functional impact, sleep quality, and selected intracranial pulse waveform parameters after the combined intervention. Given the exploratory design, these observations support further controlled investigation rather than conclusions regarding treatment efficacy or systemic neurophysiological modulation.

The results should be interpreted as initial proof‐of‐concept evidence, indicating a consistent physiological and clinical signal that justifies future investigations in controlled studies with greater statistical power.

Several limitations should be considered. The small sample (*n* = 20), absence of randomization, blinding, and a sham‐treated control group, and lack of separate photobiomodulation‐only and ultrasound‐only groups limit causal interpretation and preclude claims of synergy. Healthy participants served as physiological comparators rather than untreated controls. The FIQ was also administered to healthy participants, although it was developed for people with fibromyalgia, which limits interpretation of those scores outside the target population. Intracranial waveform morphology provides indirect physiological information and cannot diagnose intracranial hypertension; cerebrospinal fluid opening pressure, optic nerve sheath diameter, cerebral perfusion, and autonomic or biochemical mediators were not measured. Longer‐term follow‐up and larger, appropriately controlled studies are needed to evaluate the reproducibility, clinical relevance, and potential mechanisms of the observed changes.

## 5 Conclusion

The present study demonstrated that patients with fibromyalgia exhibited altered intracranial compliance patterns when compared with healthy individuals, supporting the hypothesis that alterations in intracranial dynamics may be associated with the pathophysiology of chronic pain and central sensitization. The synergistic intervention combining photobiomodulation and therapeutic ultrasound promoted measurable physiological and clinical effects, including reduction of altered intracranial compliance patterns, improvement in the *time to peak* parameter, pain reduction, improved sleep quality, and enhancement of overall functionality.

Although the P2/P1 ratio did not demonstrate statistically significant differences after intervention, the combined analysis of waveform morphology and additional physiological parameters suggests a preliminary signal of intracranial compliance modulation following therapy. These findings reinforce the potential of noninvasive intracranial compliance monitoring as a complementary functional biomarker in fibromyalgia.

From a translational perspective, the integration of photobiomodulation and therapeutic ultrasound represents a promising biophotonic strategy capable of promoting systemic neurophysiological modulation through a noninvasive and clinically applicable approach. The convergence between objective physiological findings and subjective clinical improvements strengthens the consistency of the observed effects and reinforces the relevance of multimodal monitoring in chronic pain conditions.

Despite the limitations inherent to the exploratory pilot design, including the small sample size, absence of randomization, and lack of a placebo‐controlled group, the results provide an important proof‐of‐concept foundation for future controlled and randomized studies with greater statistical power and longitudinal follow‐up. Collectively, the findings suggest that intracranial compliance assessment may emerge as a relevant complementary tool for physiological characterization and therapeutic monitoring in fibromyalgia.

## Funding

This work was supported by Fundação de Amparo à Pesquisa do Estado de São Paulo (CEPOF 2013/0707276‐1), the Conselho Nacional de Desenvolvimento Científico e Tecnológico (INCT 465360/2014‐9), and the Cancer Prevention and Research Institute of Texas (RR220054).

## Conflicts of Interest

The authors declare no conflicts of interest.

## Supporting information


**File S1:** jbio70390‐sup‐0001‐Supplementary_File_S1.docx.

**File S2:** jbio70390‐sup‐0002‐Supplementary_File_S2.docx.

## Data Availability

The data that support the findings of this study are available on request from the corresponding author. The data are not publicly available due to privacy or ethical restrictions.
