# Supplementary material for: Noninvasive Intracranial Compliance Monitoring Following Hand‐Applied Photobiomodulation and Ultrasound in Fibromyalgia: A Pilot Study
Source: J Biophotonics. 2026 Nov 10;19(11):e70390. doi: 10.1002/jbio.70390 (PMC13750874; doi:10.1002/jbio.70390)
Supplement: Supplementary file 1 [file JBIO-19-e70390-s001.docx]

Artigo Original

Original Article

Validação da Versão Brasileira do *Fibromyalgia Impact Questionnaire* (FIQ)

Validation of the Brazilian Version of the Fibromyalgia Impact Questionnaire (FIQ)

Amélia Pasqual Marques(1), Adriana M. Barsante Santos(1*), Ana Assumpção(1), Luciana Akemi Matsutani(2), Lais V. Lage(3), Carlos Alberto B. Pereira(4)

# RESUMO

**Objetivo:** Desenvolver uma versão transcultural do *Fibromyalgia Impact Questionnaire* (FIQ) para a população brasileira e analisar sua validade e eficácia quando aplicado em pacientes com fibromialgia. **Pacientes e Métodos:** Participaram do estudo 44 pacientes com fibromialgia (FM), diagnosticados segundo os critérios do *American College of Rheumatology* (ACR), 1990. Baseados nas orientações de Guillemin *et al*(22), foram convidados quatro professores de língua inglesa, um reumatologista e dois fisioterapeutas. O procedimento seguiu as etapas: tradução inicial por dois professores de inglês, avaliação das duas traduções para uma versão única, versão para a língua inglesa por dois professores de inglês nativos, reunião de consenso com dois professores de inglês, reumatologista e fisioterapeutas para versão teste, avaliação da equivalência cultural, versão final, avaliação da confiabilidade e reprodutibilidade. A versão teste foi aplicada em 20 pacientes com FM, tendo em todas as questões o item “não-aplicável”. Substituição de possíveis questões com mais de 15% de respostas “não-aplicável” por outras de mesmo conceito, resultando na versão final. Aplicação dessa versão em 24 pacientes com FM por dois avaliadores que fizeram a entrevista no mesmo dia com intervalo de uma hora e, após um período de sete dias da primeira avaliação, o questionário foi reaplicado pelo primeiro avaliador. **Resultados:** Na aplicação da versão teste não houve questões com mais de 15% de respostas “não-aplicável”. Sendo assim, não foi mudado o texto para a versão final. Porém, houve dificuldade de compreensão das escalas visuais analógicas (questões 4 a 10). Com isso, foram acrescidas “carinhas” nos dois extremos: à esquerda uma “carinha” feliz e à direita, uma infeliz. Esse processo deu

*ABSTRACT*

***Objective:*** *To develop a cross-cultural validation of the Fibromy-algia Impact Questionnaire (FIQ) to the Brazilian population and to analyze its validity and efficacy when applied to fibromyalgia patients.* ***Patients and Methods:*** *Forty-four fibromyalgia patients classified following the 1990 American College Rheumatol-ogy (ACR) criteria. According to the 1993 Guillemin et al(22) guidelines, four English teachers, one rheumatologist and two physical therapist were invited to participate. The procedure was established according to the following steps: initial translation by two English teachers, evaluation of these two translations to a unique version, back translation to English by two native teachers, a consensus meeting where there were two English teachers, one rheumatologist and physiotherapists for the test-translation, evaluation of cultural equivalency, final version, reli-ability and reproducibility. The test-translation has been applied to 20 fibromyalgia patients, having in all questions the item “not applicable”. Questions answered as “not applicable” by 15% or more patients were reformulated, resulting in the final version. Application of the final version to 24 fibromyalgia patients by two different interviewers who applied the final questionnaire in the same day, with one hour interval and seven days after the first interview, the questionnaire was re-applied by the first interviewer.* ***Results:*** *During the application of the test-version none of the questions were answered as “not applicable” for 15% or more. Consequently, the text was not reformulated. However, there were some difficulty for understanding the visual analogic scales*

Departamento de Fisioterapia, Fonoaudiologia e Terapia Ocupacional da Faculdade de Medicina da Universidade de São Paulo (USP). Recebido em 13/12/05. Aprovado, após revisão, em 10/02/06.

1. Departamento de Fisioterapia, Fonoaudiologia e Terapia Ocupacional da Faculdade de Medicina da USP.
2. Departamento de Fisioterapia do Centro Universitário da Fundação Instituto de Ensino para Osasco (UNIFIEO).
3. Disciplina de Reumatologia da Faculdade de Medicina da USP.
4. Professor de Estatística, Instituto de Matemática e Estatística da USP.

*Apoio: Fundação de Amparo à Pesquisa do Estado de São Paulo (FAPESP) – Bolsa de Iniciação Científica.

*Endereço para correspondência:* Amélia Pasqual Marques, Departamento de Fisioterapia, Fonoaudiologia e Terapia Ocupacional da Faculdade de Medicina da USP, Rua Cipotânea, 51, Cidade Universitária, CEP 05360-130, São Paulo, SP, Brasil, telefone: (11) 3091-7451, e-mail: [pasqual@usp.br](mailto:pasqual@usp.br)

origem à versão final. Na avaliação da confiabilidade, os resultados do avaliador 1 (primeira e segunda aplicação) e do avaliador 2 foram comparados, bem como as duas aplicações do avaliador

1. Ambas demonstraram que não houve diferença entre os dois avaliadores no que diz respeito a aplicação do questionário. A comparação entre os avaliadores foi feita com base nas médias globais. Os valores de p foram todos superiores a 10%, indicando que não houve evidência de diferenças significantes entre aplicações dos questionários nos mesmos pacientes. **Conclusão:** A versão brasileira do FIQ, o QIF, mostrou ser um instrumento válido e confiável para medir a capacidade funcional e o estado de saúde de pacientes brasileiros com FM.

**Palavras–chave:** fibromialgia, validação, questionário, FIQ.

*(questions 4 to 10). As a result, “small faces” were added in both ends: a “happy face” to the left and a “sad face” to the right. The evaluation of reliability and reproducibility obtained by the first interviewer (between the first and second application) as well as results obtained by the second interviewer were compared. Both showed no difference among the two interviewers in what concerns the questionnaire application. The p values were above 10% indicating no significant difference between questionnaires application.* ***Conclusion:*** *The FIQ Brazilian version, QIF, showed to be a reliable and valid instrument to measure the functional ability and the health state of Brazilian fibromyalgia patients.*

***Keywords:*** *fibromyalgia, validation, questionnaire, FIQ.*

## InTRODUÇÃO

Fibromialgia (FM) é uma síndrome complexa de ori-gem desconhecida, caracterizada por dor difusa e crônica, presença de *tender points* e, freqüentemente, associada à fadiga, ansiedade, distúrbios do sono e incapacidade fun-cional(1).

A utilização de questionários de avaliação da qualidade de vida tem sido reconhecida como uma importante área do conhecimento científico no campo da saúde, uma vez que permite uma avaliação mais objetiva de sintomas tão subjetivos, como dor, ansiedade, depressão, entre outros. Na prática clínica, esses podem identificar as necessidades dos pacientes e avaliar (estimar, calcular, dimensionar) a efetividade da intervenção. Em experimentos clínicos con-trolados, servem como instrumento de medida dos resulta-dos e são também um importante componente da análise de custo-utilidade do tratamento(2). Segundo Treurniet *et al*(3), os protocolos devem estar aptos a detectar variações de saúde entre dois grupos, variações ao longo do tempo, sendo um aspecto fundamental no registro hospitalar dos pacientes, assumindo um grande papel nos estudos expe-rimentais, prospectivos e retrospectivos.

Neste sentido, é importante que o instrumento seja amplamente adaptado às características socioculturais da população a ser analisada, permitindo uma avaliação fidedig-na da população. Além disso, é preciso que o instrumento seja facilmente administrado e que não exija muito tempo na sua aplicação. Com essa adaptação sociocultural esses objetivos acabam sendo também alcançados.

Em 1991, Burckhardt *et al*(4) propuseram e testaram um

instrumento para avaliação da qualidade de vida específico para FM, o *Fibromyalgia Impact Questionnaire* (FIQ). Este questionário envolve questões relacionadas à capacidade funcional, situação profissional, distúrbios psicológicos e sintomas físicos. É composto por 19 questões, organizadas em 10 itens. Quanto maior o escore, maior é o impacto da fibromialgia na qualidade de vida. Os autores concluíram que o FIQ é válido para ser utilizado em situações clínicas e de pesquisa. Diversos estudos subseqüentes, com pacientes com FM, utilizaram este instrumento e verificaram através dele o impacto negativo causado pela FM(5-14).

O FIQ foi traduzido e validado para muitos idiomas. Entre eles estão a versão hebraica(15), alemã(16), turca(17), coreana(18), espanhola(19), italiana(20) e francesa(21).

No Brasil, ainda não há uma proposta do FIQ validada. Embora amplamente utilizado, existem apenas traduções do referido instrumento, porém sem a preocupação de haver uma adaptação para a cultura brasileira.

As adaptações socioculturais são de fundamental im-portância para que se possa aproveitar todas as questões do instrumento, visando a análise da qualidade de vida em pacientes com FM, podendo ser utilizada como auxílio no diagnóstico e tratamento da doença.

# OBJETIVO

Desenvolver uma versão transcultural do FIQ e avaliar sua confiabilidade e validade para ser usado em pacientes brasileiros com FM.

## PACIEnTES E MÉTODOS

### Participantes

Participaram do estudo 44 indivíduos com FM, diagnosticados por reumatologista, segundo os critérios do *American College of Rheumatology* (ACR)(1), quatro professores de língua inglesa, um reumatologista e dois fisioterapeutas. Houve solicitação de autorização à autora do FIQ e obtivemos a concordância para realizar a tradução. O tamanho da amostra de n = 44 deveu-se à disponibilidade do tempo dos pacientes e das pesquisadoras. Note que o foco não foi a estimativa de parâmetros populacionais, mas apenas o poder de reprodutibilidade do FIQ em sua versão brasileira.

### Procedimento

A tradução para a língua portuguesa foi realizada se-guindo as etapas propostas por Guillemin(22):

### Tradução inicial

Os itens da versão do FIQ foram inicialmente traduzi-dos do inglês para a língua portuguesa por dois professores de inglês independentes, brasileiros, cientes da finalidade da pesquisa. Além da tradução literária, foi enfatizada a tradu-ção conceitual. As duas traduções foram comparadas pelos tradutores, pelo pesquisador e pelo orientador da pesquisa, chegando a um consenso de uma versão em português.

### Avaliação da tradução inicial

A versão em português, denominada como “Ques-tionário sobre o Impacto da Fibromialgia” (QIF), obtida na tradução inicial, foi submetida à versão para a língua inglesa por dois professores de inglês, nativos, que não haviam participado anteriormente. Em seguida, foi feita a comparação da versão para o inglês e da tradução inicial com o questionário do FIQ original. Esta comparação foi feita por uma banca composta por: um reumatologista, dois professores de inglês que tinham participado da tradução inicial e da avaliação da tradução inicial e dois fisioterapeu-tas. Esse processo resultou na versão em português.

### Avaliação da equivalência cultural

Como o FIQ origina-se da língua inglesa, com ênfase no inglês americano e nas questões próprias da cultura, foi necessário realizar a equivalência cultural para utilizá-lo em nossa população. Este aspecto foi de grande relevância para que realmente fossem avaliadas atividades realizadas

pela população brasileira.

A cada uma das questões da versão em português, foi acrescido o item “não-aplicável” e tinha como objetivo identificar as questões não compatíveis, não compreendidas ou não executadas pela população entrevistada.

Essa versão com a opção “não-aplicável” - a versão teste - foi aplicada em 20 pacientes com FM, encaminha-dos por reumatologista e selecionados aleatoriamente no Ambulatório de Reumatologia do Hospital das Clinicas da Faculdade de Medicina da Universidade de São Paulo (USP). Substituição de possíveis questões com mais de 15% de respostas “não-aplicável” por outras de mesmo conceito, resultando na versão final.

### Avaliação da confiabilidade e reprodutibilidade

A reprodutibilidade foi realizada com a versão final do QIF para a língua portuguesa. Foi aplicada a 24 pacientes com FM por dois fisioterapeutas, que fizeram a entrevista no mesmo dia com intervalo de uma hora. Após um perí-odo de sete dias da primeira avaliação, o questionário foi reaplicado pelo primeiro avaliador. Durante este período não houve qualquer mudança de conduta.

### Validade

Para analisar a validade de conteúdo, ou seja, quais itens do FIQ produziram informações úteis para os pacientes com FM, identificamos a porcentagem de indivíduos que responderam cada um dos dez itens da função física com respostas denotando comprometimento físico. Foi critério de corte ter 25% de comprometimento em cada item váli-do(16).

## AnÁLISE ESTATÍSTICA

Para fins de análise estatística, os dez itens do QIF foram normalizados de forma a variarem em uma escala de 0 a 10. Como resultado global de um questionário, consideramos duas medidas, a primeira, M1, considerando apenas a média dos sete itens com medidas contínuas que se situam no intervalo de 0 a 10 pontos. A segunda média global, M2, correspondente à média dos dez itens do FIQ. Os dados foram todos normalizados para variarem de 0 a 10 obtendo-se o escore total. Cada item possui pontua-ções diferentes (o item capacidade funcional foi somado e dividido por 30, os dois itens de 0 a 7 divididos por sete e os itens de 0 a 10 divididos por 10).

Os resultados do avaliador 1 na primeira e segunda aplicações e do segundo avaliador foram comparados. Também foi utilizado o teste de hipótese.

No teste de hipótese, apenas as médias globais (Tabela 4) foram comparadas por meio de um teste t-Student pa-reado. A comparação de cada item por meio dessa técnica não parece ser adequada, em razão dos diversos empates de medida. Considerando as médias globais, os empates não ocorrem com tanta freqüência. O nível de significância foi p≤ 0,05.

# RESULTADOS

Na aplicação da versão teste não houve questões com mais de 15% de respostas “não-aplicável”. Sendo assim, não foi mudado o texto para a versão final. Porém, houve dificuldade de compreensão da escala visual analógica (ques-tões 4 a 10). Com isso, foram acrescidas “carinhas” nos dois extremos: à esquerda uma “carinha” feliz e à direita, uma infeliz, originando a versão final.

A média, desvio padrão, e valores mínimos e máximos dos dados demográficos dos 44 pacientes participantes (20 na versão teste e 24 na versão final) estão representados na Tabela 1.

dos sete itens com medidas contínuas que se situam no intervalo de 0 a 10 pontos, quanto de M2, que corres-ponde à média dos dez itens do QIF.

Tabela 2

Média (m) e Erro-Padrão (EP)

DA VERSÃO FINAL, POR ITEM

Tabela 1

| **Avaliador 1** | | | **Avaliador 2** | | **Avaliador 1**  **(após 7 dias)** | |
| --- | --- | --- | --- | --- | --- | --- |
| **Amostra n=** | **24** |  | **24** |  | **22** | |
| **Parâmetro** | **M** | **EP** | **M** | **EP** | **M** | **EP** |
| Capacidade Funcional | 10,58 | 1,46 | 10,04 | 1,61 | 11,50 | 1,60 |
| Sentiu-se Bem | 1,79 | 0,43 | 2,13 | 0,41 | 1,73 | 0,32 |
| Faltas ao Trabalho | 1,21 | 0,33 | 1,79 | 0,39 | 2,23 | 0,53 |
| Capacidade de Trabalhar | 7,53 | 0,43 | 8,13 | 0,37 | 7,26 | 0,63 |
| Dor | 8,40 | 0,41 | 8,50 | 0,34 | 8,73 | 0,32 |
| Fadiga | 8,28 | 0,42 | 7,80 | 0,49 | 7,70 | 0,60 |
| Cansaço Matinal | 7,60 | 0,83 | 7,45 | 0,80 | 7,90 | 0,85 |
| Rigidez | 7,70 | 0,36 | 7,71 | 0,23 | 7,90 | 0,68 |
| Ansiedade | 7,45 | 0,38 | 7,95 | 0,19 | 7,60 | 0,38 |
| Depressão | 8,23 | 0,16 | 8,37 | 0,12 | 7,77 | 0,35 |
| **M1** | 7,88 | 0,29 | 7,99 | 0,37 | 7,84 | 0,44 |
| **M2** | 6,30 | 0,22 | 6,51 | 0,29 | 6,43 | 0,36 |

Dados demográficos das amostras na aplicação da versão teste e da versão final

| **Aplicação da versão teste (n=20)** | | | **Aplicação da versão final (n=24)** | |
| --- | --- | --- | --- | --- |
| **Gênero** | **95% fem** | | **100% fem** | |
|  | **Média ± DP** | **Mín-Máx** | **Média ± DP** | **Mín-Máx** |
| **Idade (anos)** | 46,15 ± 10 | 36-65 | 50,45 ±  10,28 | 28-64 |
| **Peso (kg)** | 66,86 ±  14,14 | 44-98 | 66,49 ±  8,73 | 51- 86,40 |
| **Altura (m)** | 1,57 ± 0,08 | 1,46-1,69 | 162,5 ±  0,06 | 1,52-1,72 |
| **IMC (Kg/m^2^)** | 27,76 ±  4,14 | 17,85  - 41,32 | 25,83 ±  4,23 | 18,42  - 32,54 |
| **Escolaridade (anos)** | 8,15 ± 4,15 | 3-16 | 7,00 ± 4,89 | 0-16 |

A Tabela 2 apresenta os resultados médios e os er-ros-padrão correspondentes à aplicação da versão final realizada pelos dois avaliadores. Como resultado global de um questionário, consideramos as duas medidas. Há uma clara indicação de que não houve diferença entre os dois avaliadores no que diz respeito à aplicação do questionário, tanto nos valores de M1, que considerou apenas a média

A Tabela 3 apresenta as médias e os erros-padrão das di-ferenças entre as três aplicações da versão final. Os resultados do avaliador 1 (primeira e segunda aplicações) e do avaliador 2 foram comparados bem como as duas aplicações do ava-liador 1. A exemplo do observado na Tabela 3, há indicação clara de que não houve diferença entre os dois avaliadores no que diz respeito à aplicação do questionário.

A Tabela 4 apresenta a comparação entre os avaliadores com base nas médias globais. Os valores de p são todos superiores a 10%, indicando que não há evidência de dife-renças significantes entre as aplicações dos questionários nos mesmos pacientes.

# DISCUSSÃO

Devido à natureza multifatorial da FM, faz-se neces-sária a utilização de questionários para uma avaliação mais objetiva dos sintomas subjetivos, auxiliando o diagnóstico e tratamento dessa síndrome. O FIQ é o instrumento mais

utilizado para avaliar o impacto da FM e já foi traduzido para vários idiomas.

Sem uma adaptação sociocultural, a análise dos pacien-tes com FM se apresenta deficiente, pois muitas questões acabam não sendo discriminantes.

No caso da versão turca, por exemplo, as questões como o número de dias que faltou ao trabalho (*How many days in the past week did you miss work because*

Tabela 3

Média (M) e Erro-Padrão (EP) da diferença entre pesquisadores

*your fibromyalgia?*) e a questão que envolve a habilidade de trabalhar (*When you did go to work, how much did pain or other symptoms of your fibromyalgia interfere with your ability to do your job?*) tiveram muitas respostas em branco, pois na Turquia é baixo o índice de mulheres que trabalham. Além disso, questões como trabalhar no jardim (*do yard work*) e dirigir carro (*drive a car*) também não foram muito aplicáveis na Turquia, pois a maioria das pessoas mora em apartamento e grande parte das mulhe-res não dirige carros. Porém, os pesquisadores optaram por nada fazer por considerar que esses itens não seriam

**Avaliador 1 com Avaliador 2 (aplicação no mesmo dia)**

**Intra-avaliador 1 (intervalo de 7 dias de aplicação)**

**Avaliador 2 com a 2ª aplicação do Avaliador 1**

**(após 7 dias)**

diferenciadores, já que tanto mulheres com FM quanto o

grupo-controle estariam na mesma situação.

No caso da versão coreana, os pesquisadores se depara-ram com outras questões de acordo com a realidade socioe-conômica do país. Por exemplo, a questão que versava sobre arrumar as camas (*make beds*) foi substituída por preparar o

**Amostra n=	24	24	22**

**Parâmetro	M	EP	M	EP	M	EP**

Capacidade Funcional

-0,25	1,46	-0,50	1,61	-0,14	1,60

Sentiu-se Bem	-0,33	0,43	0,14	0,41	0,50	0,32

Faltas ao Trabalho

-0,58	0,33	-0,91	0,39	-0,27	0,53

colchão (*prepare a mattress*) pelo fato de na Coréia não ser comum o uso de camas. A questão que se referia sobre andar vários quarteirões (*walk several blocks*) foi substituída por an-dar mais que um quilômetro (*walk more than one kilomete*), pois os pesquisadores concluíram que assim haveria maior compreensão das pessoas. A questão referente a trabalhar no

Capacidade de

Trabalhar

Dor	-0,10	0,41	-0,43	0,34	-0,28	0,32

-0,61	0,43	0,20	0,37	0,87	0,63

jardim (*do yard work*) também teve problemas na validação da versão coreana, pelo mesmo fato de a maioria das casas na Coréia não possuir jardins. A questão referente a dirigir

Fadiga	0,48	0,42	0,45	0,49	0,02	0,60

Cansaço Matinal

0,15	0,83	-0,09	0,80	-0,11	0,85

Rigidez	-0,01	0,36	-0,02	0,23	-0,01	0,68

Ansiedade	-0,51	0,38	0,00	0,19	0,55	0,38

Depressão	-0,14	0,16	0,39	0,12	0,58	0,35

**M1**	-0,10	0,19	0,07	0,29	0,23	0,27

**M2**	-0,21	0,16	-0,08	0,24	0,19	0,20

Tabela 4

Comparação das aplicações do questionário:

t-Student pareado

carros (*drive a car*) foi completada com andar de bicicleta (*drive a car or bicycle*), pois este transporte é comumente utilizado na Coréia.

Como pudemos observar nos estudos que realizaram a validação, é importante que além da tradução se faça a adaptação sociocultural da versão para a língua, neste caso, a portuguesa do Brasil, para que a FM possa ser melhor avaliada no país.

Alguns problemas e dificuldades já foram observados quando da aplicação do FIQ na versão traduzida e não-validada para língua portuguesa. Por exemplo, no primeiro item, os subitens “passar aspirador de pó no tapete” e “di-rigir carros”, muitas vezes não são fatores diferenciadores,

**Comparação:**

**Avaliador 1 com Avaliador 2 (aplicação no mesmo dia)**

**Intra-avaliador 1 (intervalo de 7 dias de aplicação)**

**Avaliador**

**2 com a 2ª aplicação do Avaliador 1**

**(após 7 dias)**

**Amostra n=	24	24	22**

pois as pessoas marcam que nunca realizam essas tarefas simplesmente pelo fato de não possuírem aspirador ou carro, realidade bastante comum no Brasil.

Neste trabalho, usamos as recomendações internacio-nais para validação de questionários(22).

**Parâmetro	T	pv	T	pv	T	pv**

**M1**	-0,55	58,8%	0,25	80,6%	0,86	39,7%

**M2**	-1,36	18,8%	-0,32	75,4%	0,95	35,2%

Na tradução e adaptação da versão original do FIQ, foram realizadas algumas modificações como substituição de “passar aspirador de pó” como sugere a versão original (*vacuum a rug*) para “fazer limpeza” (varrer, passar pano,

etc); inclusão de quintal junto ao jardim (*do yard work*);

“andar de ônibus” ao invés de somente “dirigir carro” (*dri-ve a car*). Embora estas mudanças impliquem em esforços e funções diferentes das propostas no FIQ original, elas foram necessárias, pois correspondem mais à realidade e às funções desempenhadas pelos pacientes com FM, uma vez que, no Brasil, muitos não dirigem ou não possuem carro, é mais comum a presença de quintal do que jardim nas casas, e uma parcela mínima da população possui aspi-rador de pó.

Também foram feitas algumas inclusões como, por exemplo, o item 0 nas questões 2 e 3 que não existiam

# CONCLUSÃO

A versão brasileira do FIQ, o QIF, mostrou ser um ins-trumento de fácil compreensão e aplicação; válido e confiável para medir a capacidade funcional e o estado de saúde de pacientes brasileiros com FM, melhor auxiliando, assim, no diagnóstico e tratamento dessa síndrome.

*The Fibromyalgia Impact Questionnaire* (FIQ)

1. **Were you able to: Always Most times Occasionally Never**

**a) Do shopping**	0	1	2	3

na versão original, pois há a possibilidade dessa resposta, já que se refere a número de dias. Também foi incluída a alternativa “trabalho em casa” na questão 3, uma vez que há pacientes que somente desenvolvem atividades do lar. Além disso, foi discutido que as pacientes afastadas do trabalho por causa da FM deveriam marcar o número sete

na questão 3, indicando o máximo de faltas no trabalho,

**b) Do laundry with**

**a washer and dryer**

**c) Prepare meals**	0	1	2	3

**d) Wash dishes/ cooking utensils by hand**

**e) Vacuum a rug**	0	1	2	3

0	1	2	3

0	1	2	3

por isso significar o máximo de incapacidade laboral. Já na questão 8, optou-se por adicionar a expressão “corpo travado” por considerá-la de mais fácil compreensão pela

**f) Make beds**	0	1	2	3

**g) Walk several blocks**

0

1

2

3

população. Além disso, foi incluído no questionário a per-gunta sobre anos de estudo, por considerarmos relevante na compreensão das perguntas.

**i) Do yard work**	0	1	2	3

**h) Visit friends / relatives**

0	1	2	3

Na aplicação da versão teste do QIF, não houve mais de 15% de respostas “não-aplicável”, mostrando que essa versão já apresentava um nível de compreensão bastante alto, não sendo preciso, portanto, modificar qualquer alter-nativa. Porém, foi observada dificuldade de compreensão das escalas visuais do QIF, provavelmente devido ao baixo nível de escolaridade da população em que foi aplicado o questionário. Com isso, à versão final foram acrescidas “carinhas” nos dois extremos: à esquerda a “carinha” feliz e à direita, uma infeliz, facilitando em muito a compreen-são das escalas e não influenciando negativamente quando considerado o nível de escolaridade do paciente avaliado. A confiabilidade intra e entre avaliadores pode ser

**j) Drive a car**	0	1	2	3

2. Of the 7 days in the past week, how many days did you feel good?

1	2	3	4	5	6	7

3. How many days in the past week did you miss work because your fibromyalgia? (If you don’t have a job outside the home, leave this item blank).

1	2	3	4	5

4. When you did go to work, how much did pain or other symptoms of your fibromyalgia interfere with your ability to do your job?

observada nas Tabelas 2 e 3, nas quais foram comparadas

as aplicações do QIF realizadas pelo avaliador 1 (primeira e segunda aplicação) e pelo avaliador 2. Há indicação clara que não houve diferença entre os dois avaliadores no que

No problem	Great difficulty

5. How bad has your pain been?

diz respeito à aplicação do questionário. Este fato é confir-

mado na Tabela 4, na qual os dados comparam os dados da aplicação entre os avaliadores com base nas médias globais. Os valores de p são todos superiores a 10%, indicando que

No pain	Very severe pain

6. How tired have you been?

não houve diferença significante entre as aplicações dos

questionários.	No tiredness	Very tired

7. How have you felt when you got up in the morning?

A woke well rested	A woke very tired

8. How bad has your stiffness been?

No stiffness	Very stiff

9. How tense, nervous or anxious have you felt?

Not tense	Very tense

10. How depressed or blue have you felt?

Not depressed	Very depressed

Questionário Sobre o Impacto da Fibromialgia (QIF)

#### Nos últimos sete dias:

2- Nos últimos sete dias, em quantos dias você se sentiu bem?

0	1	2	3	4	5	6	7

3- Por causa da fibromialgia, quantos dias você faltou ao tra-balho (ou deixou de trabalhar, se você trabalha em casa)?

0	1	2	3	4	5	6	7

4- Quanto a fibromialgia interferiu na capacidade de fazer seu serviço:

Não interferiu	Atrapalhou muito 5- Quanta dor você sentiu?

Nenhuma	Muita dor

6- Você sentiu cansaço?

**ANOS DE ESTUDO:**

| **1- Com que freqüência você consegue:** | **Sempre** | **Quase sempre** | **De vez em quando** | **Nunca** |
| --- | --- | --- | --- | --- |
| **a) Fazer compras** | 0 | 1 | 2 | 3 |
| **b) Lavar roupa** | 0 | 1 | 2 | 3 |
| **c) Cozinhar** | 0 | 1 | 2 | 3 |
| **d) Lavar louça** | 0 | 1 | 2 | 3 |
| **e) Limpar a casa**  **(varrer, passar pano etc.)** | 0 | 1 | 2 | 3 |
| **f) Arrumar a cama** | 0 | 1 | 2 | 3 |
| **g) Andar vários quarteirões** | 0 | 1 | 2 | 3 |
| **h) Visitar parentes ou amigos** | 0 | 1 | 2 | 3 |
| **i) Cuidar do quintal ou jardim** | 0 | 1 | 2 | 3 |
| **j) Dirigir carro ou andar de ônibus** | 0 | 1 | 2 | 3 |

Não	Sim, muito

7- Como você se sentiu ao se levantar de manhã?

Descansado/a	Muito cansado/a 8- Você sentiu rigidez (ou o corpo travado)?

Não	Sim, muita

9- Você se sentiu nervoso/a ou ansioso/a?

Não, nem um pouco	Sim, muito 10- Você se sentiu deprimido/a ou desanimado/a?

Não, nem um pouco	Sim, muito

**REFERÊNCIAS**

1. Wolfe F, Smythe HAA, Yunus MB, Bennett AM, Bombardier CE, Goldenberg DL: The American College of Rheumatology 1990. Criteria for the classification of fibromyalgia: Report of the Multicenter Criteria Committee. Arthritis Rheum 33: 160-72, 1990.
2. Carr AJ, Thompson PW, Kirwan JR: Quality of life measures. Br J Rheumatol 36 :275-81, 1996.
3. Treurniet HF, Essink-bot ML, Mackenbach JP, Van Der Maas PJ: Health-related quality of life: an indicator of quality of care? Qual Life Res 6: 363-9, 1997.
4. Burckardt CS, Clark SR, Bennett RM: The Fibromyalgia Impact Questionnarie: Development and Validation. J Rheumatol 18:

**30	Rev Bras Reumatol,** v. 46, n. 1, p. 24-31, jan/fev, 2006

728-33, 1991.

5. Burckhardt CS, Mannerkorpi K, Hedenberg L, Bjelle A: A randomized, controlled clinical trial of education and physical training for women with fibromyalgia. J Rheumatol 21: 714-20, 1994
6. Martinez JE, Ferraz MB, Sato EI, Atra E: Avaliação seqüencial do impacto fibromialgia e artrite reumatóide na qualidade de vida. Rev Bras Reumatol 34: 309-16, 1994.
7. Martinez JE, Ferraz MB, Sato EI, Atra E: Fibromyalgia versus rheumatoid arthritis: a longitudinal comparison of the quality of life. J Rheumatol 22: 270-4, 1995.
8. Goldenberg DL, Mossey CJ, Schmid CH: A model to assess severity and impact of fibromyalgia. J Rheumatol 22: 2313-8, 1995.
9. Bennett RM, Burckhardt CS, Clark SR, O´Reilly CA, Wiens AN, Campbell SM: Group treatment of fibromyalgia: a 6 month outpatient program. J Rheumatol 23: 521-8: 1996.
10. Gowans SE, Dehueck A, Voss S, Richardson M: A randomized, controlled trial of exercise and education for individuals with fibromyalgia. Arthritis Care Res 12: 120-8, 1999.
11. Wolfe F, Hawley DJ, Goldenberg DL, Russell IJ, Buskila D, Neumann L: The assessment of functional scales and the development of the FM Health Assessment Questionnaire. J Rheumatol 27: 1989-99, 2000.
12. Dunkl PR, Taylor AG, McConnell GG, Alfano AP, Conaway MR: Responsiveness of fibromyalgia clinical trial outcome measures. J Rheumatol 27: 2683-91, 2000.
13. Mengshoel AM, Haugen M: Health status in Fibromyalgia – a follow up study. J Rheumatol 28: 2085-9, 2001.
14. White KP, Nielson WR, Harth M, Ostbye T, Speechley M: Chronic widespread musculoskeletal pain with or without

fibromyalgia: psychological distress in a representative community adult sample. J Rheumatol 29: 588-94, 2002.

15. Buskila D, Neumann L: Assessing functional disability and health status of women with fibromyalgia: validation of a Hebrew version of the Fibromyalgia Impact Questionnaire. J Rheumatol 23: 903-6, 1996
16. Offenbaecher M, Waltz M., Schoeps P: Validation of a German version of the Fibromyalgia Impact Questionnaire (FIQ-G). J Rheumatol 27: 1984-8, 2000.
17. Sarmer S, Ergin S, Yavuzer G: The validity of the Turkish version of the Fibromyalgia Impact Questionnaire. Rheumatol Int 20: 9-12, 2000.
18. Kim, YA, Lee SS, Park K: Validation of a Korean Version of the Fibromyalgia Impact Questionnaire. J Korean Med Sci 17: 220-4, 2002.
19. García R, Guarín C, Caballero CV et al: Validación de la versión al español del Fibromyalgia Impact Questionnaire (FIQ/S) / Validation of the spanish version of the Fibromyalgia Impact Questionnnaire (FIQ). Rev Colomb Reumatol 10: 218-225, 2003.
20. Sarzi PP, Atzeni F, Fiorini T et al: Validation of an Italian version of the Fibromyalgia Impact Questionnaire (FIQ-I). Clin Exp Rheumatol 21: 459-64, 2003.
21. Perrot S, Dumont D, Guillemin F, Pouchot J, Coste J: French group for quality of life research. Quality of life in women with fibromyalgia syndrome: validation of the QIF, the French version of the Fibromyalgia Impact Questionnaire. J Rheumatol 30: 1054-9, 2003.
22. Guillemin F, Bombardier C, Beaton D: Cross-cultural adaptation of health-related quality of life measures: Literature review and proposed guidelines. J Clinical Epidemiol 46: 1417-32, 1993.
