# Supplementary material for: Noninvasive Intracranial Compliance Monitoring Following Hand‐Applied Photobiomodulation and Ultrasound in Fibromyalgia: A Pilot Study
Source: J Biophotonics. 2026 Nov 10;19(11):e70390. doi: 10.1002/jbio.70390 (PMC13750874; doi:10.1002/jbio.70390)
Supplement: Supplementary file 2 [file JBIO-19-e70390-s002.docx]

# Índice da qualidade do sono de Pittsburgh

As seguintes perguntas são relativas aos seus hábitos de sono **durante o ultimo mês somente**. Suas respostas devem indicar a lembrança mais exata da maioria dos dias e noites do ultimo mês. Por favor, responda a todas as perguntas.

Nome:

Idade:

Data:

1. Durante o último mês, quando você geralmente foi para a cama a noite? hora usual de deitar:
2. Durante o último mês, quanto tempo (em minutos) você geralmente levou para dormir a noite?

número de minutos:

3. Durante o último mês, quando você geralmente levantou de manhã? hora usual de levantar?
4. Durante o último mês, quantas horas de sono você teve por noite? (Esta pode ser diferente do número de horas que você ficou na cama)

Horas de sono por noite:

5. Durante o último mês, com que frequência você teve dificuldade para dormir porque você:
   A) não conseguiu adormecer em até 30 minutos

1 = nenhuma no último mês	2 = menos de uma vez por semana 3 = uma ou duas vezes por semana	4 = três ou mais vezes na semana

B) acordou no meio da noite ou de manhã cedo

1 = nenhuma no último mês	2 = menos de uma vez por semana 3 = uma ou duas vezes por semana	4 = três ou mais vezes na semana

C) precisou levantar para ir ao banheiro

1 = nenhuma no último mês	2 = menos de uma vez por semana 3 = uma ou duas vezes por semana	4 = três ou mais vezes na semana

D) não conseguiu respirar confortavelmente

1 = nenhuma no último mês	2 = menos de uma vez por semana 3 = uma ou duas vezes por semana	4 = três ou mais vezes na semana

E) tossiu ou roncou forte

1 = nenhuma no último mês	2 = menos de uma vez por semana 3 = uma ou duas vezes por semana	4 = três ou mais vezes na semana

F) Sentiu muito frio

1 = nenhuma no último mês	2 = menos de uma vez por semana 3 = uma ou duas vezes por semana	4 = três ou mais vezes na semana

G) sentiu muito calor

1 = nenhuma no último mês	2 = menos de uma vez por semana 3 = uma ou duas vezes por semana	4 = três ou mais vezes na semana

H) teve sonhos ruins

1 = nenhuma no último mês	2 = menos de uma vez por semana 3 = uma ou duas vezes por semana	4 = três ou mais vezes na semana

I) teve dor

1 = nenhuma no último mês	2 = menos de uma vez por semana 3 = uma ou duas vezes por semana	4 = três ou mais vezes na semana

J) outras razões, por favor descreva:	1 = nenhuma no último mês	2 = menos de uma vez por semana 3 = uma ou duas vezes por semana	4 = três ou mais vezes na semana

6. Durante o último mês como você classificaria a qualidade do seu sono de uma maneira geral:

Muito boa	Boa	Ruim	Muito ruim

7. Durante o último mês, com que frequencia você tomou medicamento (prescrito ou por conta própria) para lhe ajudar

1 = nenhuma no último mês	2 = menos de uma vez por semana 3 = uma ou duas vezes por semana	4 = três ou mais vezes na semana

8. No último mês, que frequencia você teve dificuldade para ficar acordado enquanto dirigia, comia ou participava de uma atividade social (festa, reunião de amigos)

1 = nenhuma no último mês	2 = menos de uma vez por semana 3 = uma ou duas vezes por semana	4 = três ou mais vezes na semana

9. Durante o último mês, quão problemático foi pra você manter o entusiasmo (ânimo) para fazer as coisas (suas atividades habituais)?

Nenhuma dificuldade	Um problema leve

Um problema razoável	Um grande problema

10. Você tem um parceiro (a), esposo (a) ou colega de quarto?
    A) Não
    B) Parceiro ou colega, mas em outro quarto
    C) Parceiro no mesmo quarto, mas em outra cama
    D) Parceiro na mesma cama

Se você tem um parceiro ou colega de quarto pergunte a ele com que freqüência, no ultimo mês você apresentou:

E) Ronco forte

1 = nenhuma no último mês	2 = menos de uma vez por semana 3 = uma ou duas vezes por semana	4 = três ou mais vezes na semana

F) Longas paradas de respiração enquanto dormia

1 = nenhuma no último mês	2 = menos de uma vez por semana 3 = uma ou duas vezes por semana	4 = três ou mais vezes na semana

G) contrações ou puxões de pernas enquanto dormia

1 = nenhuma no último mês	2 = menos de uma vez por semana 3 = uma ou duas vezes por semana	4 = três ou mais vezes na semana

D) episodios de desorientação ou confusão durante o sono

1 = nenhuma no último mês	2 = menos de uma vez por semana 3 = uma ou duas vezes por semana	4 = três ou mais vezes na semana

E) Outras alterações (inquietações) enquanto você dorme, por favor descreva:

1 = nenhuma no último mês	2 = menos de uma vez por semana 3 = uma ou duas vezes por semana	4 = três ou mais vezes na semana
